# A Metabolomic Approach for the Discrimination of Red Ginseng Root Parts and Targeted Validation

**DOI:** 10.3390/molecules22030471

**Published:** 2017-03-15

**Authors:** Gyo In, Hyun Kyu Seo, Hee-Won Park, Kyoung Hwa Jang

**Affiliations:** Korea Ginseng Research Institute, Korea Ginseng Corporation, Daejeon 305-805, Korea; 20109042@kgc.co.kr (G.I.); gakaga@kgc.co.kr (H.K.S.); 20100288@kgc.co.kr (H.-W.P.)

**Keywords:** *Panax ginseng*, red ginseng (RG), metabolomics, ultra-performance liquid chromatography quadruple time-of-flight mass spectrometry (UPLC-QToF-MS)

## Abstract

Ginsenosides are used as existing markers of red ginseng (RG) quality, and ginsenoside ratios are also indicative of the different components of red ginseng. For the analysis and classification of ginsenoside content, red ginseng was separated into three parts, namely, main roots, lateral roots, and fine roots, and each extract was subjected to ultra-performance liquid chromatography quadruple time-of-flight mass spectrometry (UPLC-QToF-MS) with multivariate statistical analysis. Principal component analysis (PCA) showed a clear discrimination between the extracts of main roots and fine roots and suggested discrimination markers (four for the main roots and five for the fine roots). The fine root markers were identified as ginsenoside. We identified two markers for the main roots of red ginseng in this study. Moreover, the contents of 22 ginsenosides were analyzed in all three components of red ginseng. Fine roots have the highest protopanaxadiol (PPD)/protopanaxatriol (PPT) ratio. The PPD group of ginsenosides, which is quantitatively dominant in fine roots, clearly distinguishes the main roots from the other parts.

## 1. Introduction

*Panax ginseng* C.A. Meyer is one of the most widely used and valuable medicinal herbs in the world [1,2,3]. Traditionally, the root of *P. ginseng*, the most used part, is physically subdivided into three groups, namely, main roots, lateral roots, and fine roots [1]. The chemical constituents and efficacy of each of these parts has already been reported [4]. Red ginseng (the steamed roots of *P. ginseng*) is also one of the most studied medicinal plants and has been found to contain metabolites similar to those in ginseng, such as ginsenosides, polysaccharides, and lipids [1,4]. Among these, ginsenosides, which are representative metabolites, have been the target of useful research [5,6,7]. Ginsenosides are a special group of triterpenoid saponins that can be classified into two groups by the skeleton of their aglycones, dammarane- and oleanane-type. Dammarane-type ginsenosides are mainly classified according to their genuine aglycone moieties, protopanaxadiol (PPD) and protopanaxatriol (PPT) [1]. The differentiation of the amounts of ginsenosides in each part has also been reported, showing that fine roots, compared to main roots, have a 6- to 10-fold higher amount of total ginsenoside [1,8,9]. However, more ginsenosides do not always imply greater biological efficacy. Souza et al. studied the postprandial glucose (PPG) lowering efficacy of *P. ginseng* using its main root and rootlet fraction. In this study, despite a reduced ginsenoside profile, the main root extract caused a greater reduction of PPG levels than did the rootlet extract [10]. However, this study did not describe the difference between the compositions of the main roots and those of the rootlet fractions of ginseng. 

Herein, we describe the distinguishing marker components of the main roots and fine roots of red ginseng (RG) through a metabolomic approach using ultra-performance liquid chromatography with quadrupole time-of-flight mass spectrometry (UPLC-QToF-MS), and determine the ginsenoside content of each part. After non-targeted global analysis, targeted validation was performed for the identified potential marker ions. 

Metabolomic analysis was performed to distinguish each part of red ginseng, using UPLC-QToF-MS with multivariate statistical analysis. Principal component analysis (PCA) showed a clear discrimination between the extracts of main roots and fine roots, and suggested discriminating markers (four for the main roots and five for the fine roots). The fine root markers were identified as ginsenoside Rd (ion **a**), Rc (ion **b**), Rb2 (ion **c**), Rb1 (ion **d**), and 20(*S*)-Rg2 (ion **f**). On the other hand, markers for the fine roots were not ginsenosides. We tried to identify all of the markers of the main roots and were able to confirm the marker ion **g** in gingerglycolipid B via comparison with another publication [11]. Additionally, marker ion **l** was 13-*cis*-docosenamide, which was the same compound as that found in our previous report [12]. The others (ion **h** and **k**), owing to their lower amounts, did not result in anything.

Meanwhile, the amounts of 22 ginsenosides (NG-R1, Ra1, Rb1, Rb2, Rb3, Rc, Rd, Re, Rf, Rg1, 20(*S*)-Rg2, 20(*R*)-Rg2, 20(*S*)-Rg3, 20(*R*)-Rg3, Rg5, Rg6, 20(*S*)-Rh1, Rh4, Rk1, Rk3, Ro, and F4) were analyzed within each of the three parts (main root, lateral roots, and fine roots) of red ginseng. They were classified according to their genuine aglycone moieties such as protopanaxadiol (Rb1, Rc, Ra1, Rb2, Rb3, Rd, 20(*S*)-Rg3, 20(*R*)-Rg3, Rk1, and Rg5), protopanaxatriol (NG-R1, Rg1, Re, Rf, 20(*S*)-Rh1, 20(*S*)-Rg2, 20(*R*)-Rg2, Rg6, Rk3, F4, and Rh4), and octillol (Ro). The results showed that fine roots had the highest PPD/PPT ratio. The PPD group of ginsenosides was quantitatively dominant in fine roots, clearly distinguishing it from main roots. These results are definitely helpful for the quality control and standardization of commercial products that use red ginseng and provide a scientific basis for the pharmacological research of red ginseng.

## 2. Results and Discussion

### 2.1. Non-Targeted Component Analysis

**Figure 1:**
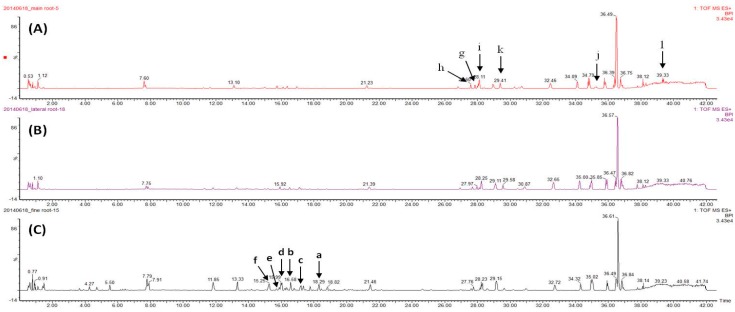
Base peak ion count chromatogram for the three components of red ginseng: (**A**): main roots; (**B**): lateral roots; (**C**): fine roots.

The three parts of 20 RG specimens were analyzed using UPLC-QToF MS. The non-targeted global analysis data were used to obtain information about potential markers for the various parts of RG. The chromatograms for the parts of RG were generated with an analysis time of 43 min, as conducted in our previous research [13]. The gradient elution mode was employed in the UPLC system to acquire the maximized chromatographic performance, including simultaneous data acquisition and appropriate retention times and integration values. It shows the total ion count chromatograms (TIC) of the main root, lateral root, and fine root samples in the given UPLC conditions (Figure 1). Figure 1 were extracted for multivariate analysis, and the accurate mass measurement was established by the simultaneous and independent acquisition of reference ions of leucine-enkephalin (*m/z* 556.2771) via the LockSpray™ interface.

**Figure 2:**
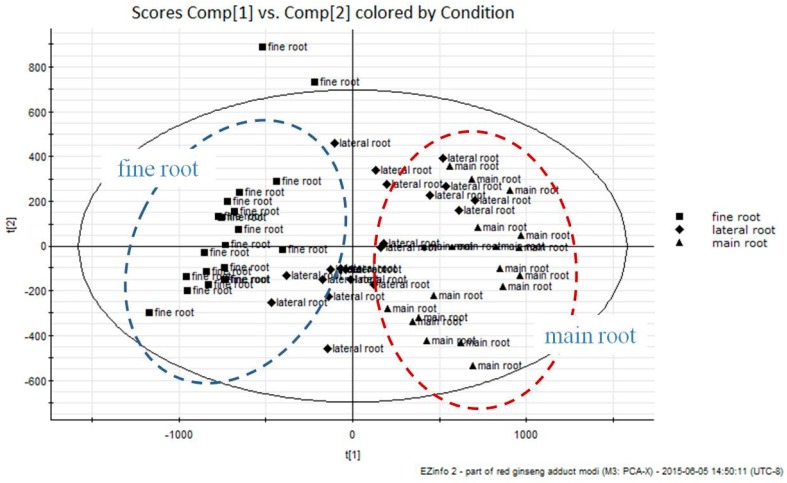
Principal component analysis (PCA) score plot for main roots and fine roots.

To find novel marker substances, unsupervised PCA and supervised OPLS-DA were performed using the non-targeted analysis data of UPLC-QToF MS (Figures S13 and S14). After creating a process for mean-centering and Pareto scaling, data were displayed as PCA score plots (Figure 2). As shown, each part of the RG specimens were very clearly clustered into three groups, namely, main roots, lateral roots, and fine roots. Thus, to explore the potential markers that contributed most to the observed differences between the different parts of RG, UPLC-QToF MS data from the main root and fine root groups were processed via supervised OPLS-DA.

**Figure 3:**
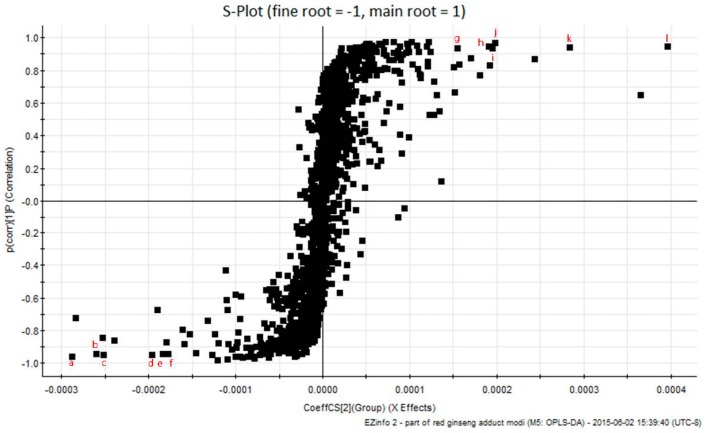
OPLS-DA S-plot between main root and fine root, using Pareto scaling with mean centering.

As shown in Figure 3, the first six ions, **a** (t_R_ 18.26 min, *m/z* 425.3798), **b** (t_R_ 16.52 min, *m/z* 425.3784), **c** (t_R_ 17.13 min, *m/z* 407.3690), **d** (t_R_ 15.94 min, *m/z* 425.3777), **e** (t_R_ 15.83 min, *m/z* 805.4426), and **f** (t_R_ 15.18 min, *m/z* 423.3632) at the lower left of the “S-curve” were the ions from the fine roots, which contributed most to the differences between the two parts of RG. The plots of ion intensity trends show that, in all samples, there were a relatively high amount of these ions in the main root samples, but these ions were undetectable or present in very small amounts in the fine root samples (Figure 3). Analogously, the six ions—**g** (t_R_ 27.97 min, *m/z* 701.3853), **h** (t_R_ 27.72 min, *m/z* 520.3447), **i** (t_R_ 28.23 min, *m/z* 542.3270), **j** (t_R_ 35.45 min, *m/z* 352.3225), **k** (t_R_ 29.60 min, *m/z* 496.3431), and **l** (t_R_ 39.33 min, *m/z* 338.3435)—at the top right corner of the “S-curve” were those from the main root samples and contributed most to the differences between the two groups. The intensities of these ions (Ions **a**–**f**) were relatively higher in all fine root samples, but were much lower in the main root. These ion intensity trends suggest that components related to Ions **a**–**f** and **g**–**l** could be used as potential chemical markers of the fine root and the main root, respectively (Figures S1~S12).

Before identification of these potential marker ions, we tried to exclude false-positive ions and fragment ions from these potential markers. Thus, the individual potential marker ions were compared with each other using their extracted chromatograms and mass spectra. The individual chromatograms and mass spectra of the selected ions are shown in the supporting information files. Two ions (Ions **e** and **j**) were found to be false-positives because the observed signal did not result from a single compound. Additionally, Potential Marker Ion **i** was a fragment ion of the same molecule (Figures S5, S9 and S10). The remaining promising results are summarized in Table 1. According to the result of the analysis of UPLC-QToF MS, nine potential markers were extracted from the parts of RG. In the case of the fine root samples, five marker ions were confirmed to be ginsenoside (**a**, ginsenoside Rd; **b**, ginsenoside Rc; **c**, ginsenoside Rb2; **d**, ginsenoside Rb1; **f**, ginsenoside 20(*S*)-Rg2). Moreover, Marker Ion **l** was 13-*cis*-docosenamide, which was identified as a marker for six-year-old RG in our previous research [12]. Isolation and spectroscopic identification were performed to identify Remaining Marker Ions **g**, **h**, and **k**.

### 2.2. Targeted Validation of Ginsenosides

To confirm the potential marker substances of the fine root samples, targeted validation was performed by the simultaneous quantification of ginsenosides in the RG samples in the same manner as our previous study [13]. As shown in Table 2, the ginsenoside content of the parts of RG are summarized in this experiment. The ginsenosides, except for Ro, were found in significantly higher amounts in the fine root samples than in those of the main roots and the lateral roots. The ginsenoside content gradually increased from the main body to the lateral roots and then to the fine roots. This ginsenoside content demonstrated the ratio of the amount of protopanaxadiol (PPD) type- to the amount of protopanaxatriol (PPT) type-ginsenoside. The ratio of PPD to PPT was 1.30 ± 0.10 for the main root samples.

**Figure 4:**
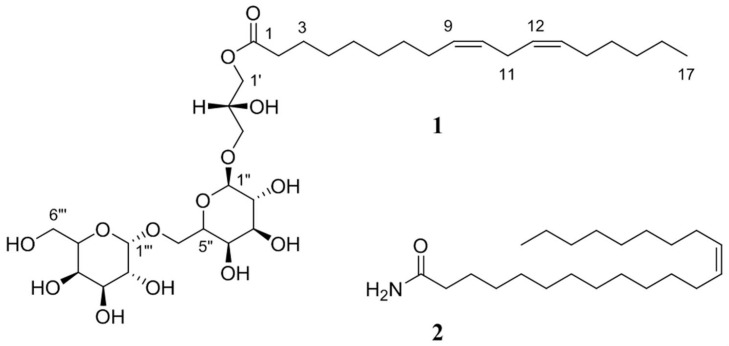
Isolated compounds from the main root of red ginseng.

To isolate the selected markers, six-year-old red ginseng was provided by the Korea Ginseng Corporation (KGC), Buyeo, Korea. The specimens were extracted twice with MeOH at 40 °C. Crude extracts were combined and fractionated by solvent partitioning. LCMS and UPLC-QToF-MS trace-guided separation of non-polar fractions was then accomplished by silica phase vacuum flash chromatography followed by silica HPLC to afford Compounds **1** and **2** (Figure 4).

Compound **1** was isolated as a white powder with the molecular formula C_33_H_58_O_14_, which was deduced from HRFABMS. The observation of a carbonyl carbon at δ_C_ 175.5 in the ^13^C-NMR spectra and an absorption band at 1760 cm^−1^ in the IR spectrum were indicative of an ester linkage. The NMR data displayed signals of 15 oxygenated carbons at δ_C_ 74.7~62.7 and their attached protons at δ_H_ 4.3~3.5. ^1^H COSY, gHSQC, and gHMBC correlations among these signals revealed the presence of a glycerol-type moiety and two sugars (Figures S15 and S16). The remaining portion of the molecule, containing the ester and all of the upfield carbons and protons, was found to form a long chain by ^1^H COSY analysis and was connected to the C-1 of glycerol, based on long-range correlations between the ester carbon and H-1 methylene protons in the gHMBC data. The linear nature of this compound was evident from the presence of several signals in the regions of δ_C_ 30.8~30.2 and δ_C_ 1.36~1.28 in the ^13^C- and ^1^H-NMR data, respectively. The fatty acid chain of **1** possessed two conjugated *cis*-double bonds, and these assignments were made based upon an analysis of the vicinal coupling constants of 10.3 and 10.5 Hz. The double bonds were placed at C-9~C-10 and C-12~C-13 by conspicuous ion clusters in FAB collision-induced dissociation (FAB-CID) MS/MS data (Figure S5). The gross structure of Compound **1** was defined as a glyceroglycolipid. Treatment of **1** with sodium methoxide in methanol furnished a glycerol digalactoside, which was identified by comparison of specific rotation (1: ${\left[\alpha \right]}_{D}^{25}$ 78.2 (*c* 0.1, H_2_O); (2’*R*)-glyceryl 6-*O*-(α-d-galactopyranosyl)-β-d-galactopyranoside: ${\left[\alpha \right]}_{D}^{25}$ 80.0 (H_2_O) and ^13^C-NMR [11,14]. Spectral data for the compound was consistent with previous report for the gingerglycolipid B [11]. Compound **2** was identified in our previous study as 13-*cis*-docosenamide [12].

The identification of **1** and **2** and its investigation as a marker for the main body of six-year-old red ginseng suggests a significant utility in the quality control and standardization of red ginseng. A more thorough examination of the mechanism of action in six-year-old red ginseng is necessary. 

## 3. Materials and Methods 

### 3.1. Plant Materials

The six-year-old RG samples used in this study were obtained from the red ginseng manufacturing factory of Korea Ginseng Corporation (Buyeo, Chung-nam, Korea). All of the 20 obtained RG were separated into three parts (main root, lateral roots, and fine roots) according to their physiological shape. The three parts of the RG were detached and dried in an oven (WiseVan, VS1202-D3, Daihan Scientific, Seoul, Korea) at 60 °C for 12 h, and all samples were immediately ground to a fine powder for analysis.

### 3.2. Chemicals and Reagents

Leucine-enkephalin was purchased from Sigma-Aldrich (St. Louis, MO, USA). Phosphoric acid was purchased from Junsei Chemical Co., Ltd (Tokyo, Japan). HPLC-grade acetonitrile and methanol were purchased from Merck (Darmstadt, Germany). All distilled water used in this experiment was purified by the Milli-Q gradient system (Millipore, Bedford, MA, USA), and the resistance was measured as 18 MΩ prior to use.

### 3.3. Sample Preparation for Metabolomics

For the UPLC-QToF-MS analysis, the powdered red ginseng samples were extracted in a manner similar to our previous studies using ultrasonic extraction [13]. In total, 300 mg of each powered red ginseng was weighed in a centrifuge tube (15 mL, PP-single use; BioLogix Group, Jinan, Shandong, China) and shaken vigorously after the addition of 6 mL of methanol. The extract was then placed in an ultrasonic cleaner (60 Hz; Wiseclean, Seoul, Korea) for 30 min. The solution was centrifuged (Legand Mach 1.6R; Thermo, Frankfurt, Germany) at a speed of 3000 rpm for 10 min, and an aliquot of the supernatant solution was filtered (0.2 µm; Acrodisk, Gelman Sciences, Ann Arbor, MI, USA) and injected into the UPLC system (Waters Co., Milford, MA, USA).

### 3.4. UPLC-QToF Analysis

The instrumental analysis was performed by ultra-performance liquid chromatography (UPLC) using an ACQUITY BEH C18 column (100 mm × 2.1 mm, 1.7 µm; Waters Co., Milford, MA, USA) on a Waters ACQUITY UPLC system with a binary solvent manager. The column temperature was 40 °C. The binary gradient elution system consisted of 0.01% formic acid in water (A) and 0.01% formic acid in acetonitrile (B). Separation was achieved using the following protocol: 0–0.5 min (15% B), 14.5 min (30% B), 15.5 min (32% B), 18.5 min (38% B), 24.0 min (43% B), 27.0 min (55% B), 27.0–31.0 min (55% B), 35.0 min (70% B), 38.0 min (90% B), 38.1 min (15% B), 38.1–43.0 min (15% B). The flow rate was 0.4 mL/min, and the sample injection volume was 2.0 µL.

Metabolite profiling of red ginseng was performed by coupling a Waters ACQUITY UPLC system to a Waters Xevo QToF mass spectrometer (Waters MS Technologies, Manchester, UK) with the positive mode of the electrospray ionization (ESI^+^) interface. The source and desolvation gas temperatures were 400 °C and 120 °C, respectively. N_2_ was used as the nebulizer and desolvation gas. The flow rates of the nebulizer gas and cone gas were set at 800 L/h and 50 L/h, respectively. The capillary and cone voltages were separately adjusted to 2150 V and 40 V. The mass accuracy and reproducibility were maintained by infusing the lockmass (leucine-enkephalin, 200 pg/L) through the Lockspray™ at a flow rate of 7 μL/min. Centroid data were collected for each sample from 150 to 1500 Da, and the *m/z* values of all acquired spectra were automatically adjusted during acquisition, based on the lockmass and dynamic range enhancement. Accurate mass and molecular formula assignments were obtained with the MassLynx™ 4.1 software (Waters MS Technologies).

### 3.5. Multivariate Analysis

To evaluate the potential characteristic components of four- and six-year-old red ginseng, the ESI^+^ raw data of all samples were calculated with the MassLynx™ application manager, version 4.1 (Waters MS Technologies, Manchester, UK). The method parameters were as follows: retention time range, 2–37 min; mass range, 150–1500 Da; mass tolerance, 0.07 Da. The peak widths at 5% height and peak-to-peak baseline noise were automatically calculated for peak integration. Additionally, the noise elimination level was set to 0.10, and the retention time tolerance was set to 0.2 min. No specific mass or adduct ions were excluded, but the isotopic peaks were removed in the multivariate analysis. For data analysis, a list of the intensities of the detected peaks was generated using the pair of retention time (t_R_) and mass data (*m/z*) as the identifier for each peak. A temporary ID was assigned to each of these t_R_-*m/z* pairs for data adjustment, based on their chromatographic elution order in UPLC. Upon completion, the correct peak intensity data for each t_R_-*m/z* pair for all samples were sorted into a table. Ions from different samples were considered to be the same when they showed identical t_R_ and *m/z* values. MarkerLynx™ (Waters MS Technologies, Manchester, UK) was used for the normalization of each detected peak against the sum of the peak intensities within that sample. The resulting data consisted of a peak number (t_R_-*m/z* pair), sample name, and ion intensity. Then, the resultant data sets were analyzed by principal component analysis (PCA) and orthogonal partial least squared discriminant analysis (OPLS-DA) using MarkerLynx™.

### 3.6. Ginsenoside Analysis

Targeted ginsenosides were analyzed in the manner to report in our previous studies [13]. In total, 0.5 g of RG powder was weighed in a centrifuge tube (15 mL, PP-single use, BioLogix, Shandong, China) and shaken vigorously after the addition of 10 mL of 70% MeOH. The extraction was performed in an ultrasonic cleaner (60 Hz, Wiseclean, Busan, Korea) for 30 min. After extraction, centrifugal separation (Legand Mach 1.6R, Thermo, Germany) was performed for 10 min at a rate of 3000. The resulting supernatant solution was filtered (0.2 µm, Acrodisk, Washington, DC, USA) and injected into the UPLC system.

The instrumental analysis was performed by a Waters ACQUITY UPLC system (Waters, Millford, MA, USA) composed of a binary solvent manager (BSM), sample manager (SM), and photo-diode array detector (PDA). The chromatographic separation program used the same conditions as those of UPLC-Q-ToF. The flow rate was set at 0.6 mL/min and the sample injection volume was 2.0 µL. Finally, the ginsenosides were detected by PDA at 203 nm. Ginsenoside Rg1, Re, Rf, 20(*S*)-Rh1, Rb1, Rc, Rb2, Rd, 20(*S*)-Rg3, and 20(*R*)-Rg3 standards were purchased from Chromadex (Irvine, KY, USA), and notoginsenoside R1, ginsenoside 20(*S*)-Rg2, 20(*R*)-Rg2, Rg6, Rk3, F4, Rh4, Ro, Rb3, Rk1, and Rg5 standards were obtained from Ambo Institute (Seoul, Korea). The method validation was performed in accordance with our previous result for the quantification of ginsenoside [15]. 

### 3.7. Isolation of Selected Markers

The six-year-old red ginseng samples (*P. ginseng*; dry weight, 5 kg) were macerated, and extracted repeatedly with MeOH (20 L × 2) at 40 °C. The combined crude extract (800 g) was partitioned between dichloromethane and H_2_O, and the dichloromethane layer (35 g) was then repartitioned between 15% aqueous MeOH (11 g) and *n*-hexane (24 g). The H_2_O layer was subjected to HP20 chromatography using sequential mixtures of MeOH and H_2_O as eluents (elution order: 100% H_2_O, 20%, 40%, 60%, and 80% MeOH in H_2_O, 100% MeOH), with a final eluent of acetone. Guided by the results of UPLC-QToF MS analyses, Fraction 6 (100% MeOH) containing secondary metabolites was combined (8.1 g), and then separated by silica vacuum flash chromatography using sequential mixtures of MeOH and dichloromethane as eluents (elution order: 100% dichloromethane, 2%, 5%, 10%, 20%, and 50% MeOH in dichloromethane, and 100% MeOH). Fraction 7 (1.1 g) was purified by silica semi-preparative HPLC (YMC ODS-A column, 1 × 25 cm, 50% aq. acetonitrile), then purified again by silica semi-preparative HPLC (YMC ODS-A column, 1 × 25 cm, 65% aq. acetonitrile) to yield 0.4 mg of Compound **1** (ion **g**) as a yellow amorphous solid.

The *n*-hexane layer was subjected to silica vacuum flash chromatography using sequential mixtures of MeOH and H_2_O as eluents (elution order: 100% *n*-hexane, 10%, 20%, 30%, 40%, 50% ethyl acetate in *n*-hexane, 100% ethyl acetate) and finally using MeOH. Guided by the results of LCMS and UPLC-QToF MS analyses, Fraction 7 containing secondary metabolites was combined (1.5 g), purified by silica semi-preparative HPLC (YMC silica column, 1 × 25 cm, 100% EtOAc), and then purified by silica semi-preparative HPLC (YMC-silica column, 5% EtOAc in *n*-hexane) to yield 0.5 mg of Compound **2** (ion **l**) as a white solid.

### 3.8. General Experimental Procedures for Compounds ***1*** and ***2***

Optical rotation was measured on a JASCO P-1020 polarimeter (Jasco, Tokyo, Japan) using a 1 cm cell. The UV spectra were recorded on a Hitachi U-3010 spectrophotometer (Hitachi High-Technologies, Tokyo, Japan), and the IR spectra were recorded on a JASCO 4200 FT-IR spectrometer (Jasco, Tokyo, Japan) using a ZnSe cell. NMR spectra were recorded in CDCl_3_ containing Me_4_Si as an internal standard on Bruker Avance 600 spectrometers (Bruker, Karlsruhe, Germany). Proton and carbon NMR spectra were measured at 600 and 150 MHz, respectively. High-resolution ESI-Q-ToF-MS/MS mass spectrometric data were obtained on an Agilent Technologies 6530 Accurate-Mass Q-ToF LC/MS spectrometer (Santa Clara, CA, USA) with an Agilent Technologies 1260 series HPLC. Low-resolution ESIMS data were recorded on an Agilent Technologies 6130 quadrupole mass spectrometer with an Agilent Technologies 1200 series HPLC. HPLC was performed on a Shimadzu LC-6AD equipped with a Shimadzu RID-10A refractive index detector (Shimadzu, Kyoto, Japan). All solvents were spectroscopic grade or distilled in a glass prior to use.

## 4. Conclusions 

Twelve differentiation markers for quality control and standardization distinguishing the main roots and fine roots of red ginseng were found using UPLC-QToF-MS. Various analytical parameters, such as the selection of ginseng, the preparation of red ginseng, and the measurement conditions of UPLC-QToF-MS, were successfully obtained. These methods were successfully applied to measure a clear discrimination between the extracts of the different components of red ginseng, and suggested a total of nine discrimination markers. All markers for fine roots are ginsenosides. These results are also supported by the targeted validation.

In the process of developing this analytical method, gingerglycolipid B (**1**) was isolated, while 13-*cis*-docosenamide (**2**) was previously defined as a marker of 6-year-old red ginseng [11,12]. The results found herein demonstrate the potential of using markers for quality control and for the standardization of the different components of red ginseng. Additionally, they throw a light on the changes in the chemical constituents of ginseng root due to the steaming process, which may be helpful for the commercial production of ginseng supplements with special chemical formulations for a variety of bodily ailments.

## Figures and Tables

**Table 1:** Selected potential markers.

	Marker Ion	Retention Time (min)	Mass to Charge Ratio (*m/z*)	Identification
Markers for fine root	Ion **a**	18.26	425.3798	Ginsenoside Rd
	Ion **b**	16.52	425.3784	Ginsenoside Rc
	Ion **c**	17.13	407.3690	Ginsenoside Rb2
	Ion **d**	15.94	425.3777	Ginsenoside Rb1
	Ion **f**	15.18	423.3632	Ginsenoside 20(*S*)-Rg2
Markers for Main root	Ion **g**	27.97	701.3853	gingerglycolipid B (**1**)
	Ion **h**	27.72	520.3447	unknown
	Ion **k**	29.60	496.3431	unknown
	Ion **l**	39.33	338.3435	13-*cis*-docosenamide (**2**)

**Table 2:** Ginsenoside content for the three components of red ginseng (RG) (*n* = 20 mg/g).

Analytes		Main Root	Lateral Roots	Fine Root
PPT ^1^-type	NG ^3^-R1	0.03 ± 0.01	0.04 ± 0.01	1.39 ± 0.10
	G ^4^-Rg1	1.85 ± 1.13	2.91 ± 1.17	4.73 ± 0.60
	G-Re	2.12 ± 1.39	2.85 ± 0.86	8.88 ± 1.04
	G-Rf	0.54 ± 0.21	0.86 ± 0.25	1.45 ± 0.18
	20(*S*)-G-Rh1	0.85 ± 0.37	0.75 ± 0.27	1.22 ± 0.31
	20(*S*)-G-Rg2	0.14 ± 0.0543	0.30 ± 0.07	0.230± 0.31
	20(*R*)-G-Rg2	0.22 ± 0.10	0.20 ± 0.09	1.52 ± 0.55
	G-Rg6	0.04 ± 0.03	0.05 ± 0.03	0.23 ± 0.04
	G-Rk3	0.08 ± 0.04	0.06 ± 0.03	0.06 ± 0.02
	G-F4	0.08 ± 0.03	0.14 ± 0.03	0.35 ± 0.06
	G-Rh4	0.17 ± 0.08	0.14 ± 0.06	0.10 ± 0.03
Octillol-type	G-Ro	0.25 ± 0.07	0.22 ± 0.05	0.14 ± 0.16
PPD ^2^-type	G-Rb1	3.89 ± 1.68	7.91 ± 2.58	20.54 ± 3.56
	G-Rc	1.15 ± 0.39	3.17 ± 0.99	9.27 ± 2.23
	G-Ra1	0.04 ± 0.16	0.07 ± 0.33	4.78 ± 3.42
	G-Rb2	1.46 ± 0.47	4.20 ± 1.28	11.33 ± 3.38
	G-Rb3	0.26 ± 0.09	0.70 ± 0.22	2.10 ± 0.57
	G-Rd	0.12 ± 0.05	0.52 ± 0.21	2.46 ± 0.60
	20(*S*)-G-Rg3	0.12 ± 0.05	0.18 ± 0.06	0.48 ± 0.07
	20(*R*)-G-Rg3	0.08 ± 0.04	0.12 ± 0.04	0.27 ± 0.05
	G-Rk1	0.07 ± 0.03	0.13 ± 0.04	0.28 ± 0.05
	G-Rg5	0.63 ± 0.121	0.98 ± 0.21	1.59 ± 0.21
sum of PPT type		6.10 ± 2.41	8.30 ± 2.41	15.90 ± 2.00
sum of PPD type		7.82 ± 2.89	17.99 ± 5.29	53.10 ± 9.12
PPD/PPT		1.30 ± 0.10	2.18 ± 0.26	3.34 ± 0.39

^1^ PPT: protopanaxatriol; ^2^ PPD: protopanaxadiol; ^3^ NG: notoginsenoside, ^4^ G: ginsenoside.

## Supplementary Materials

Supplementary materials are available online.
