# Supplementary material for: A Metabolomic Approach for the Discrimination of Red Ginseng Root Parts and Targeted Validation
Source: Molecules. 2017 Mar 15;22(3):471. doi: 10.3390/molecules22030471 (PMC6155309; doi:10.3390/molecules22030471)
Supplement: Supplementary file 1 [file molecules-22-00471-s001.pdf]

Supplementary Data

Metabolomic Approach for Discrimination of Red Ginseng Root Parts and Targeted Validation

Contents

Figure S1. Selected ion intensity trend plot and EIC for ion **a**	2
Figure S2. Selected ion intensity trend plot and EIC for ion **b**	3
Figure S3. Selected ion intensity trend plot and EIC for ion **c**	4
Figure S4. Selected ion intensity trend plot and EIC for ion **d**	5
Figure S5. Selected ion intensity trend plot and EIC for ion **e**	6
Figure S6. Selected ion intensity trend plot and EIC for ion **f**	7
Figure S7. Selected ion intensity trend plot and EIC for ion **g**	8
Figure S8. Selected ion intensity trend plot and EIC for ion **h**	9
Figure S9. Selected ion intensity trend plot and EIC for ion **i**	10
Figure S10. Selected ion intensity trend plot and EIC for ion **j**	11
Figure S11. Selected ion intensity trend plot and EIC for ion **k**	12
Figure S12. Selected ion intensity trend plot and EIC for ion **l**	13
Figure S13. PCA score plot (main root, lateral roots, and fine root)	14
Figure S14. OPLS-DA score plot (main root and fine root)	14
Figure S15. The ^{1}H NMR spectrum of compound **1** (marker 6)	15
Figure S16. The ^{13}C NMR spectrum of compound **1** (marker 6)	16

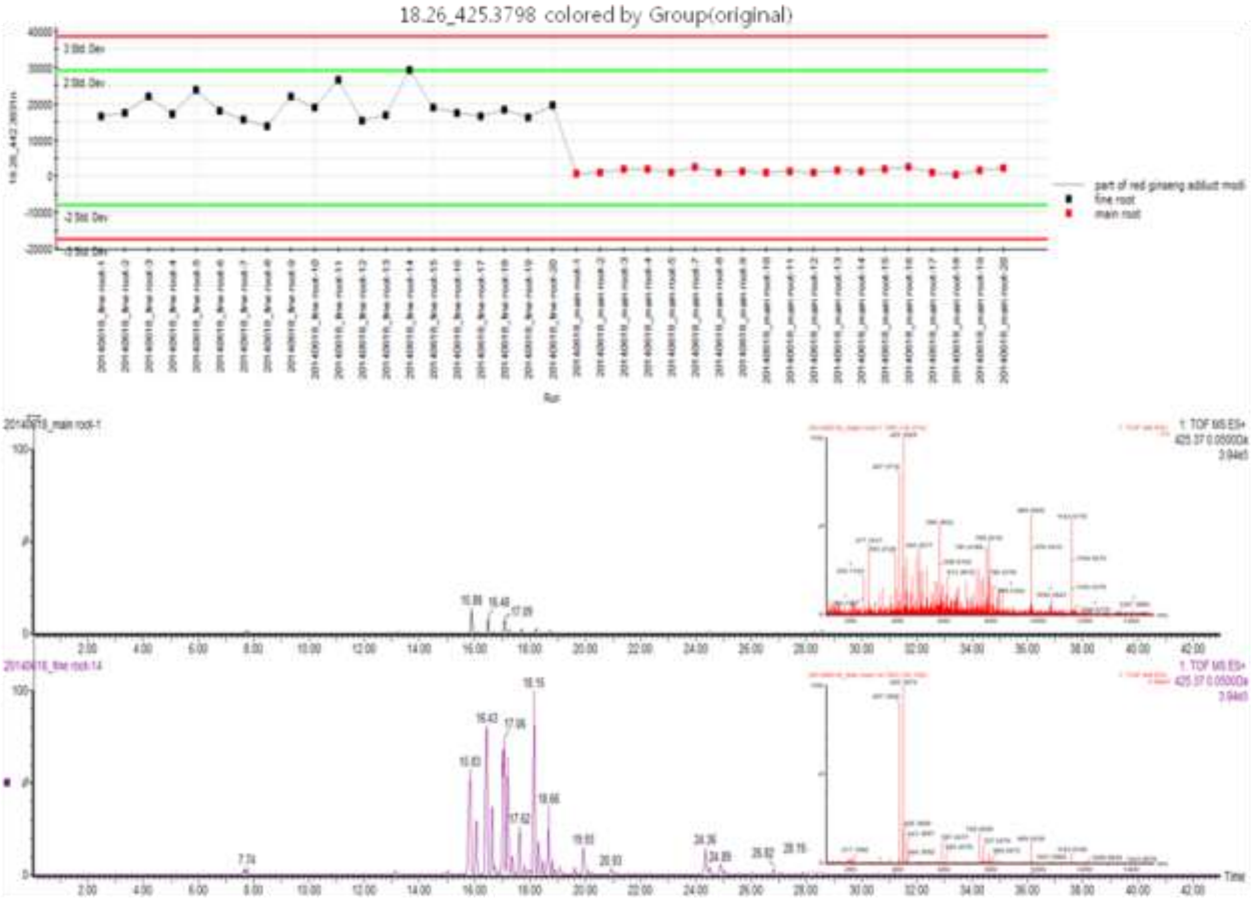

Figure S1. Selected ion intensity trend plot & EIC for ion **a**

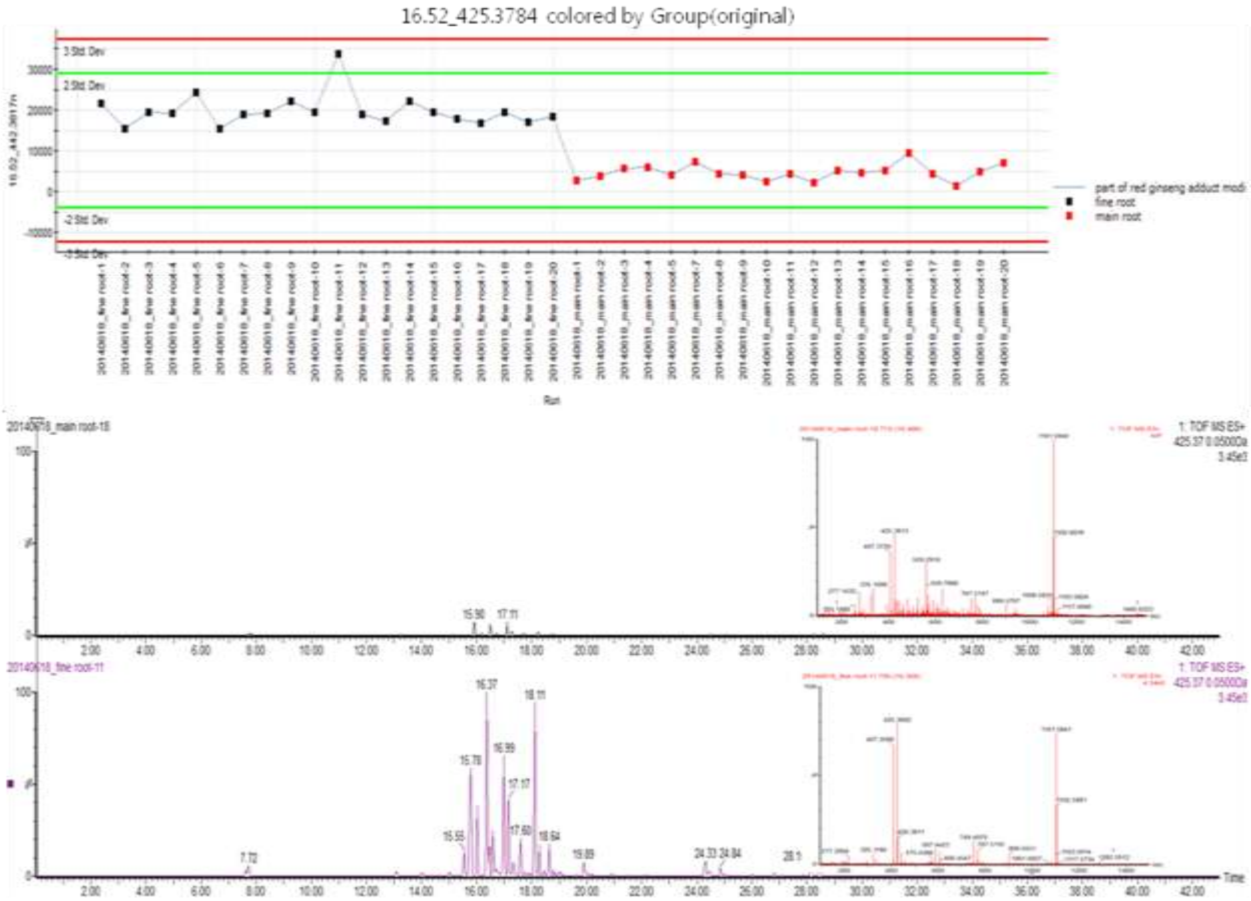

Figure S2. Selected ion intensity trend plots & EIC for ion **b**

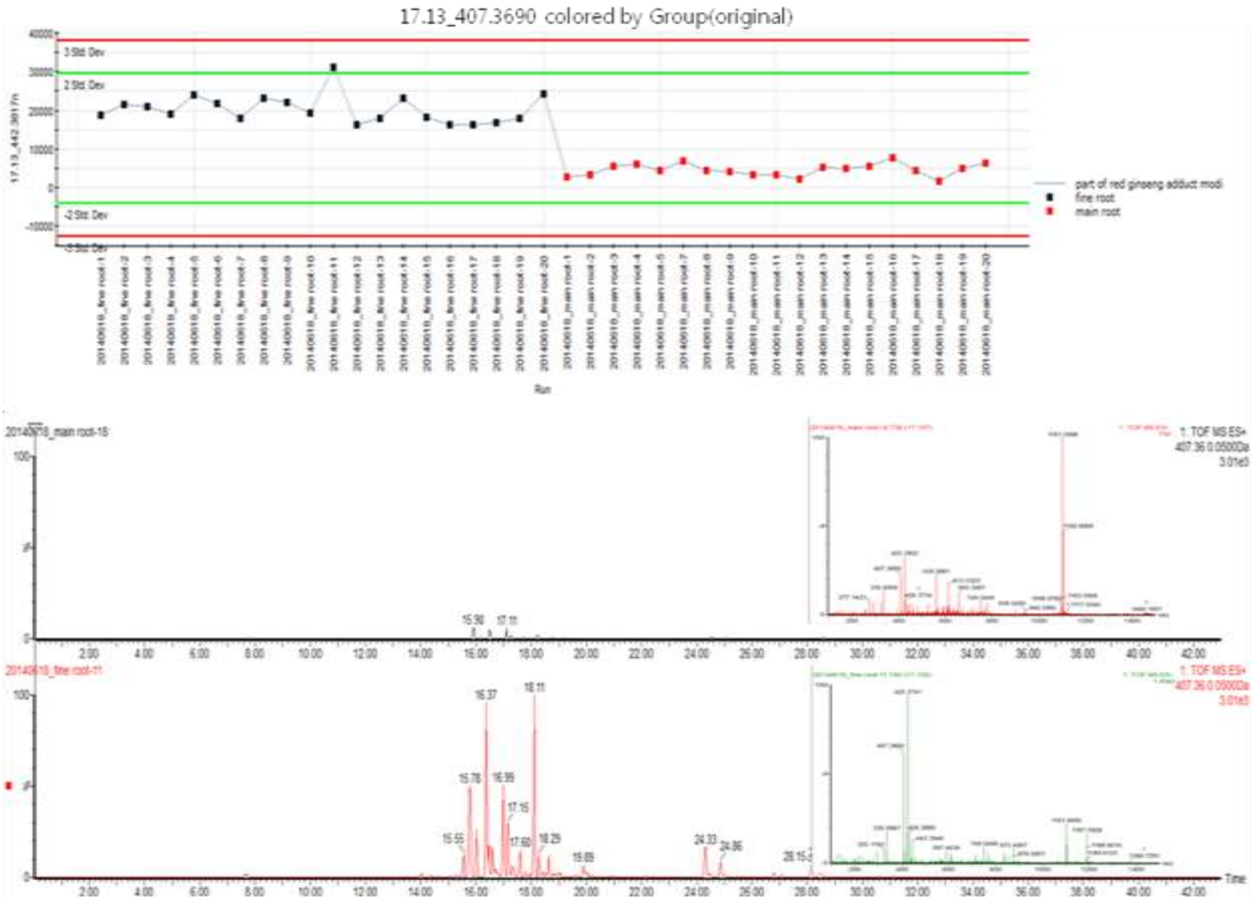

Figure S3. Selected ion intensity trend plots & EIC for ion **c**

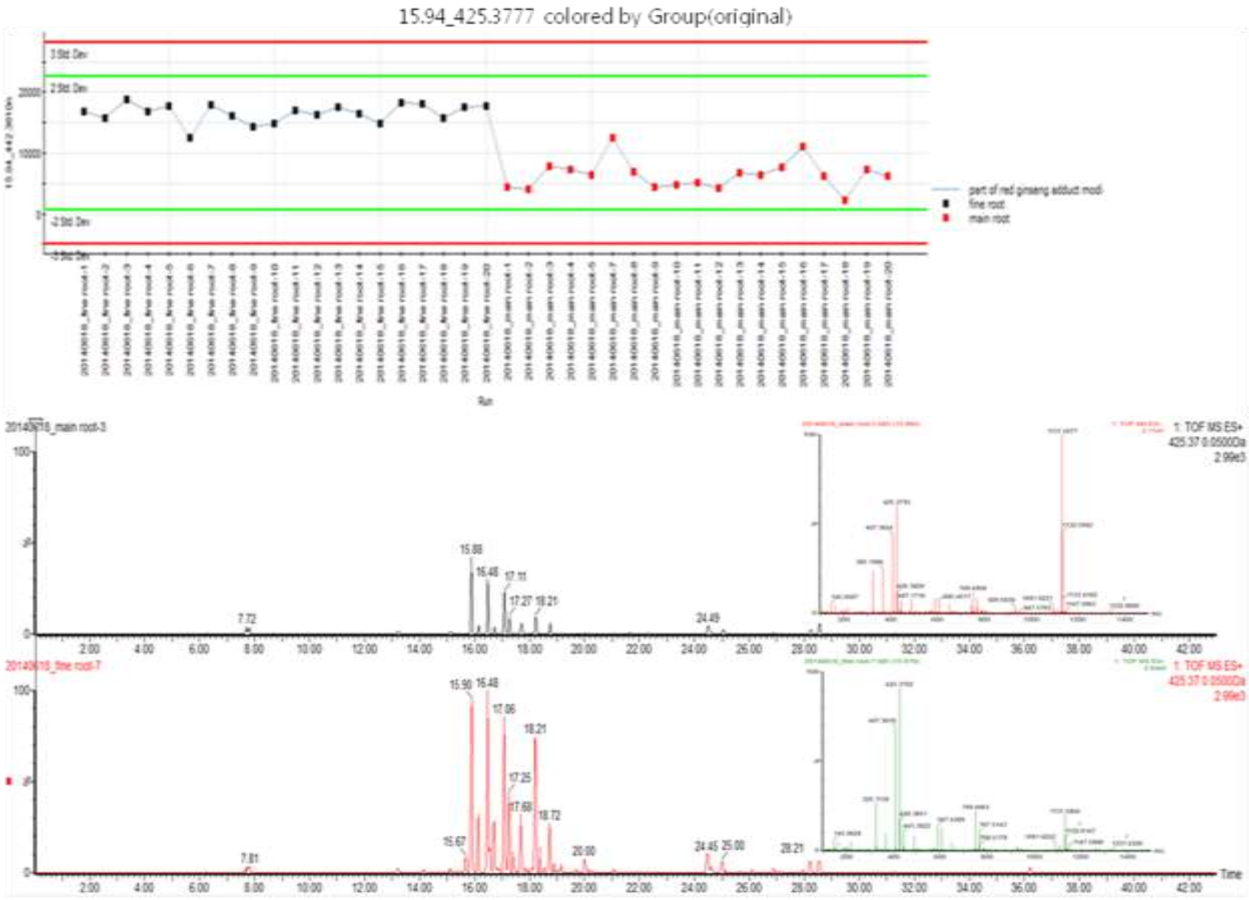

Figure S4. Selected ion intensity trend plots & EIC for ion **d**

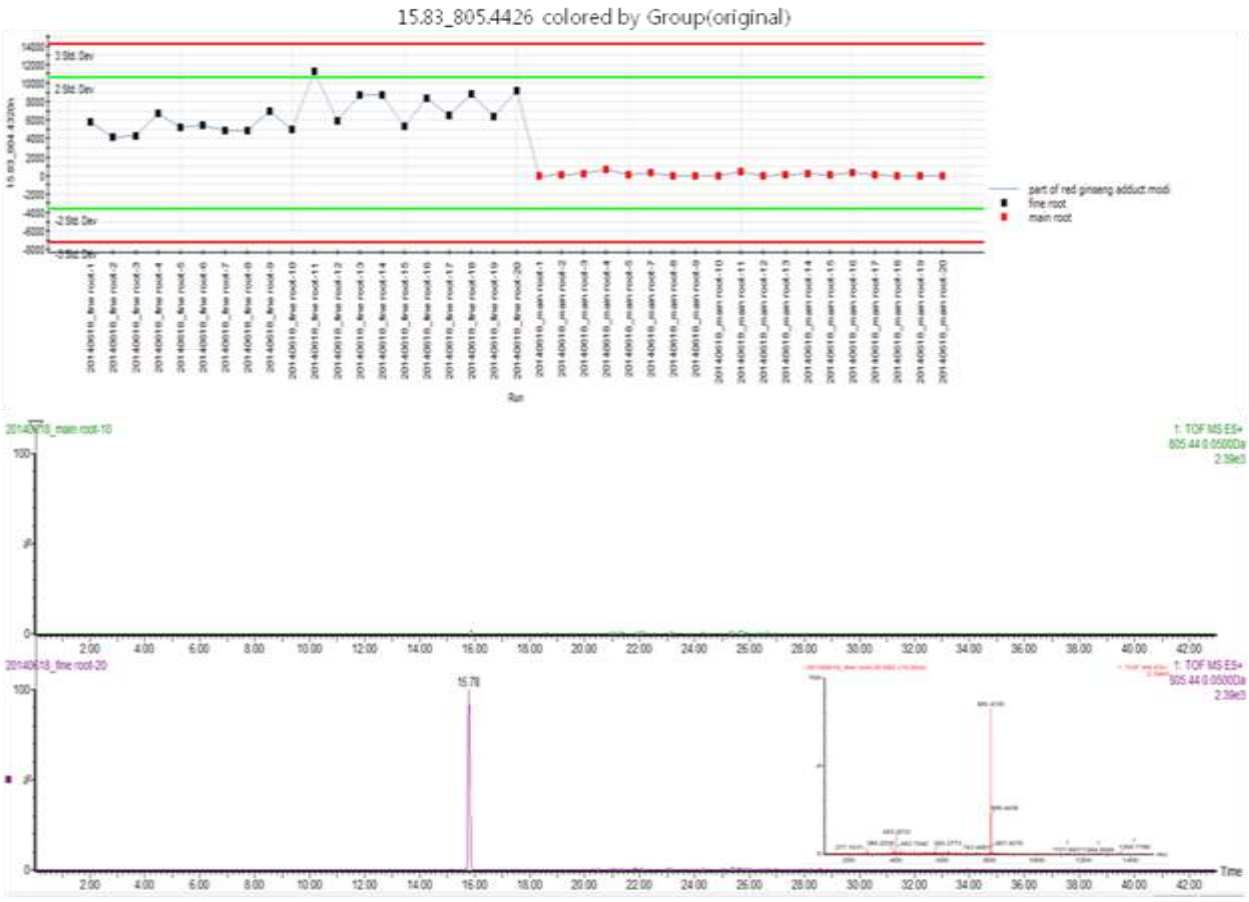

Figure S5. Selected ion intensity trend plots & EIC for ion **e**

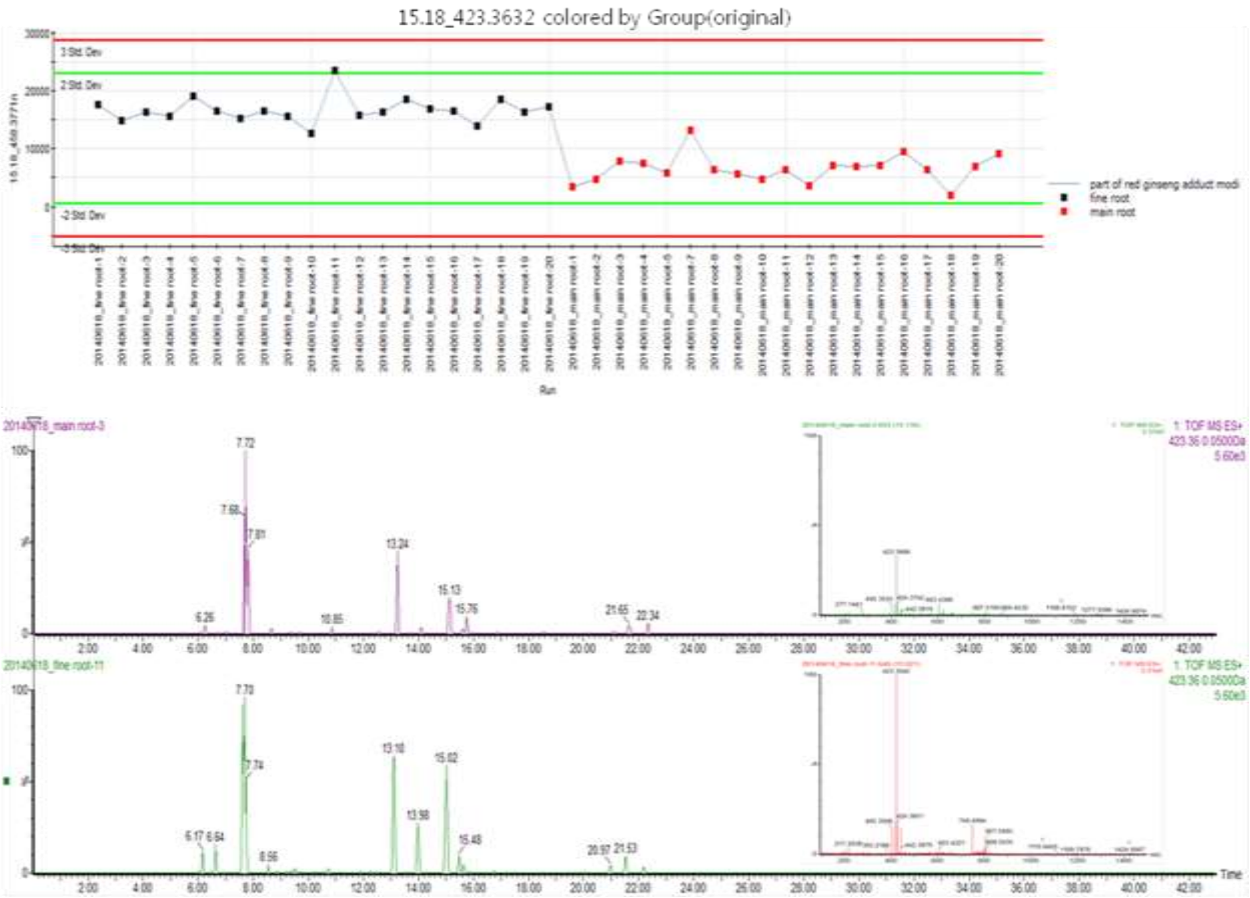

Figure S6. Selected ion intensity trend plots & EIC for ion **f**

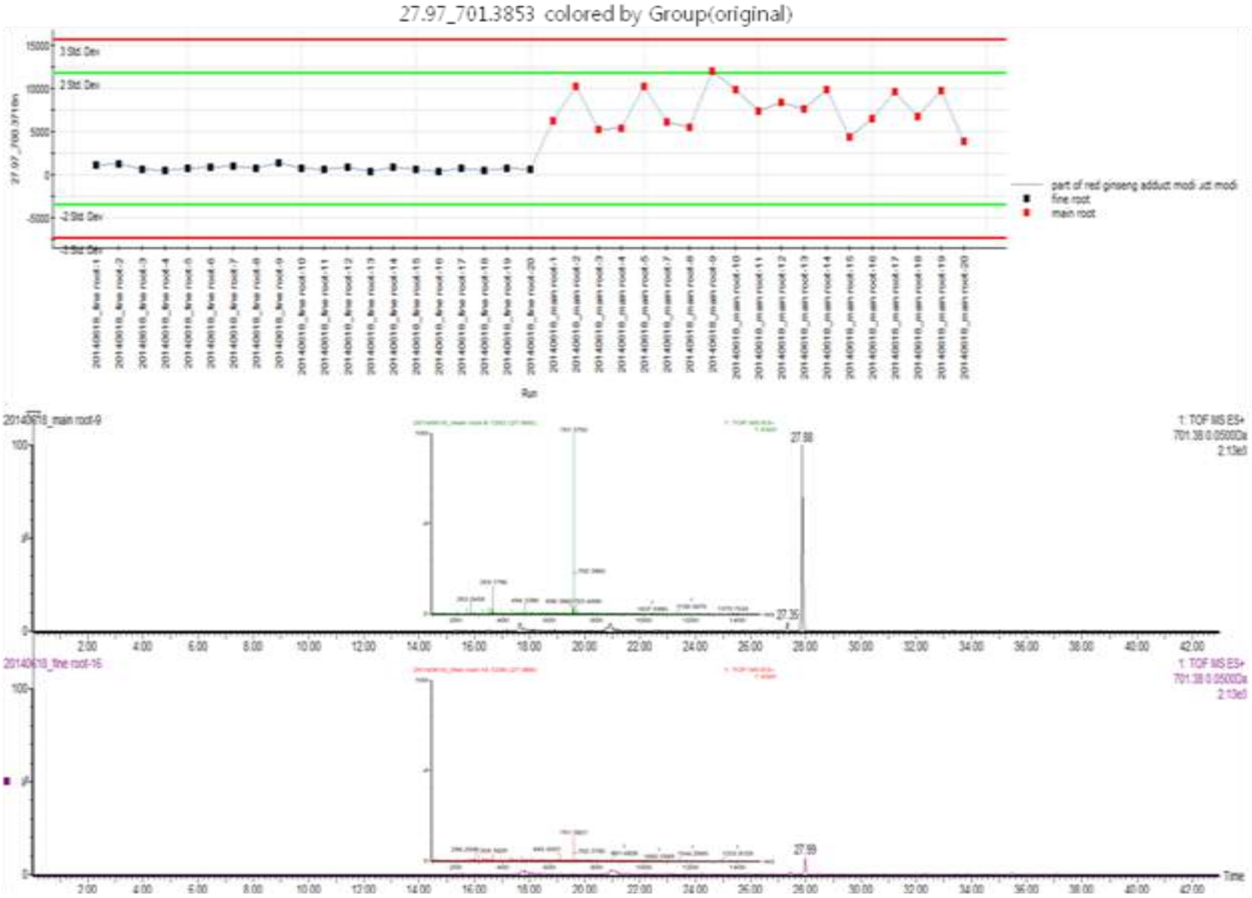

Figure S7. Selected ion intensity trend plots & EIC for ion **g**

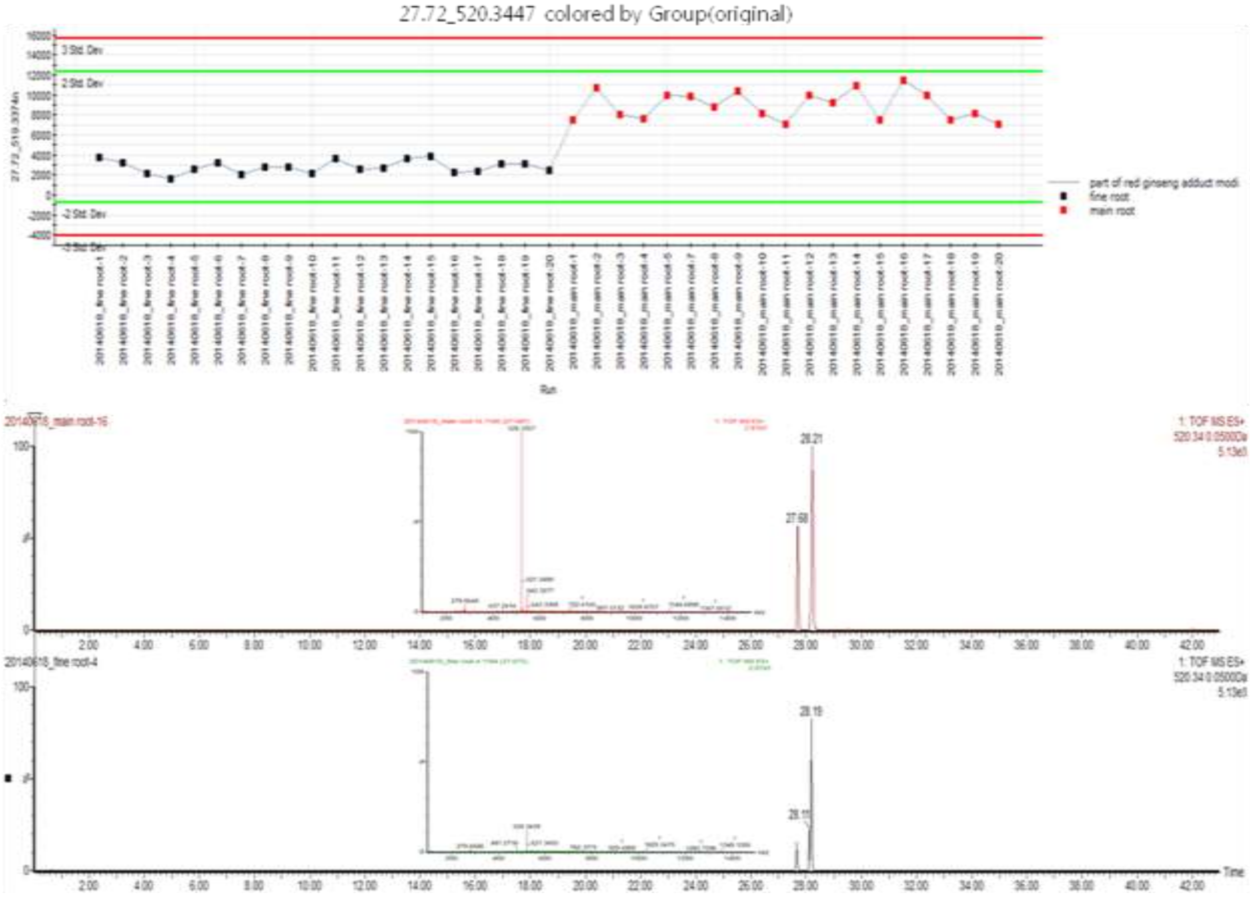

Figure S8. Selected ion intensity trend plots & EIC for ion **h**

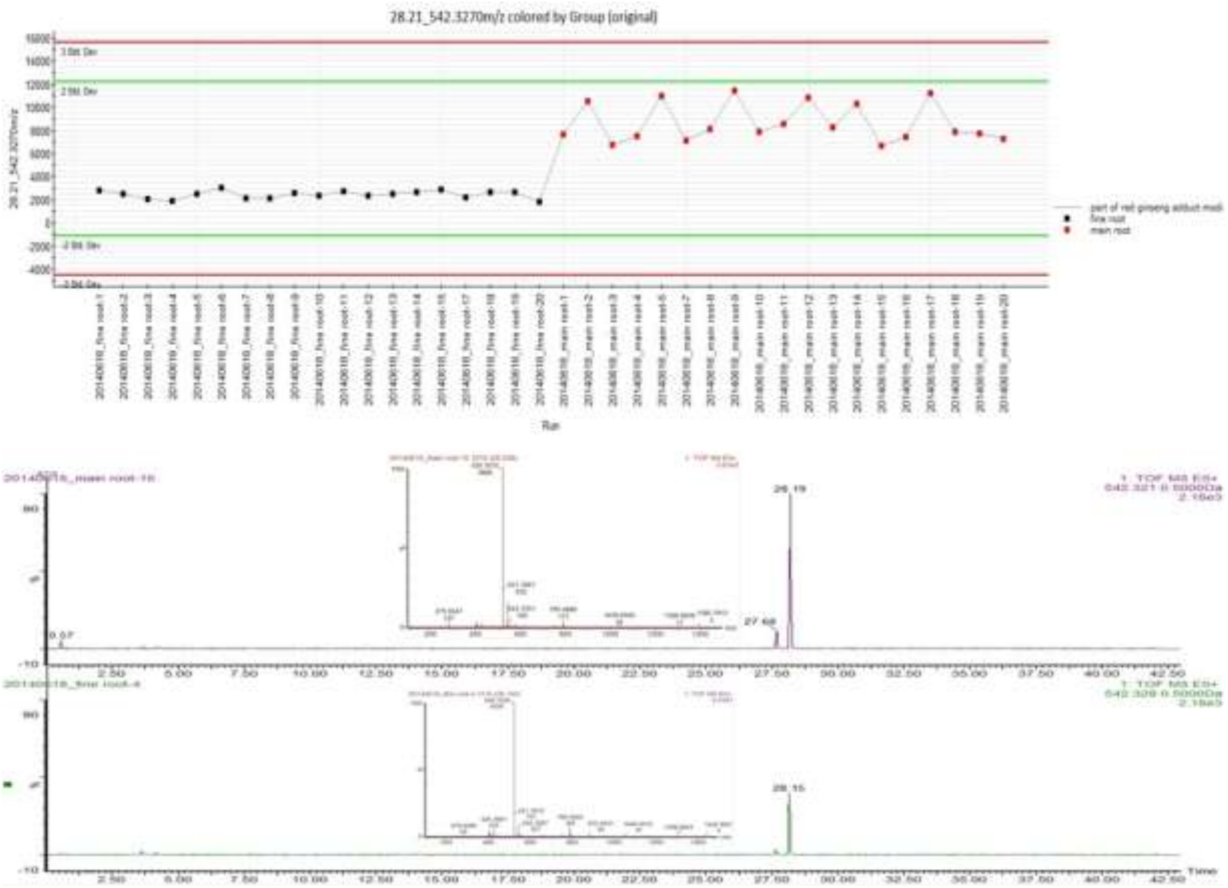

Figure S9. Selected ion intensity trend plots & EIC for ion **i**

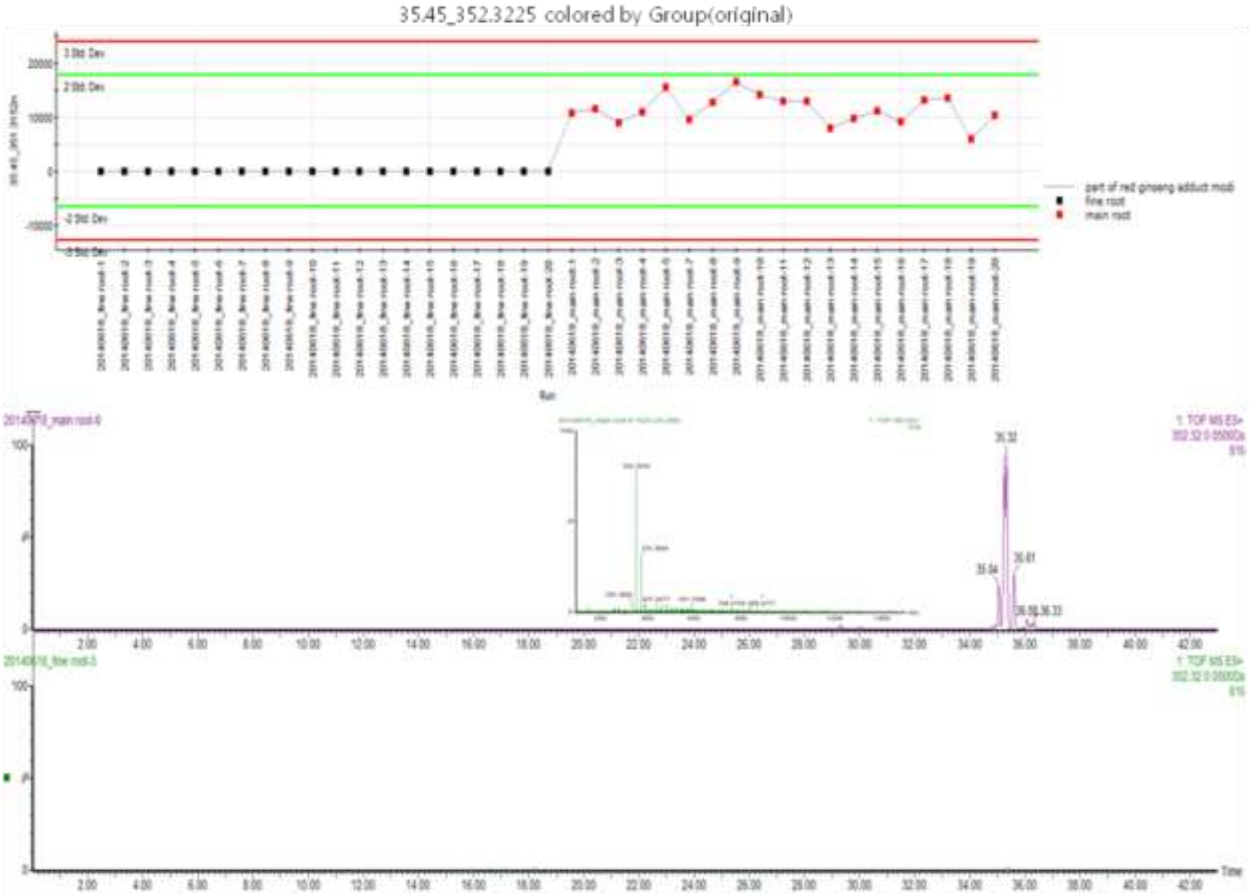

Figure S10. Selected ion intensity trend plots & EIC for ion **j**

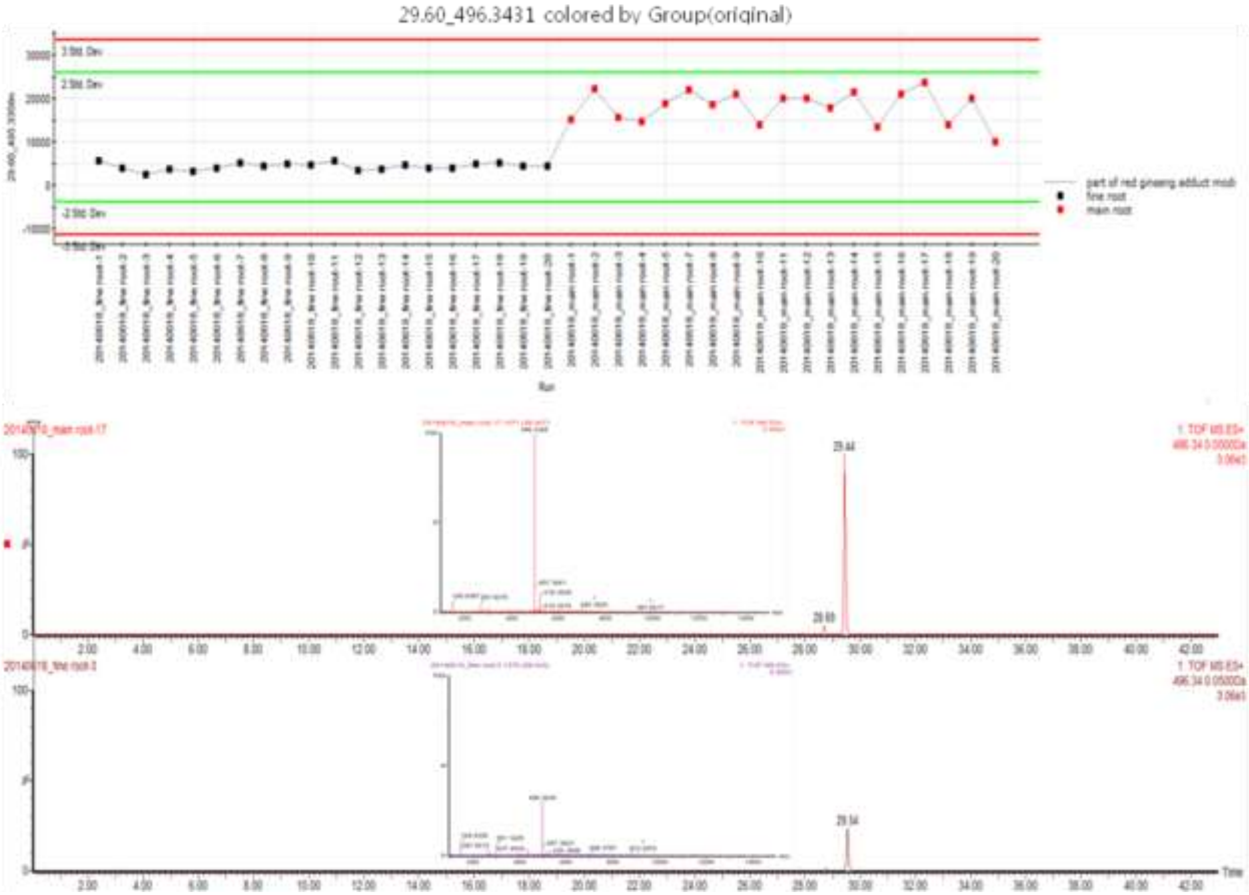

Figure S11. Selected ion intensity trend plots & EIC for ion **k**

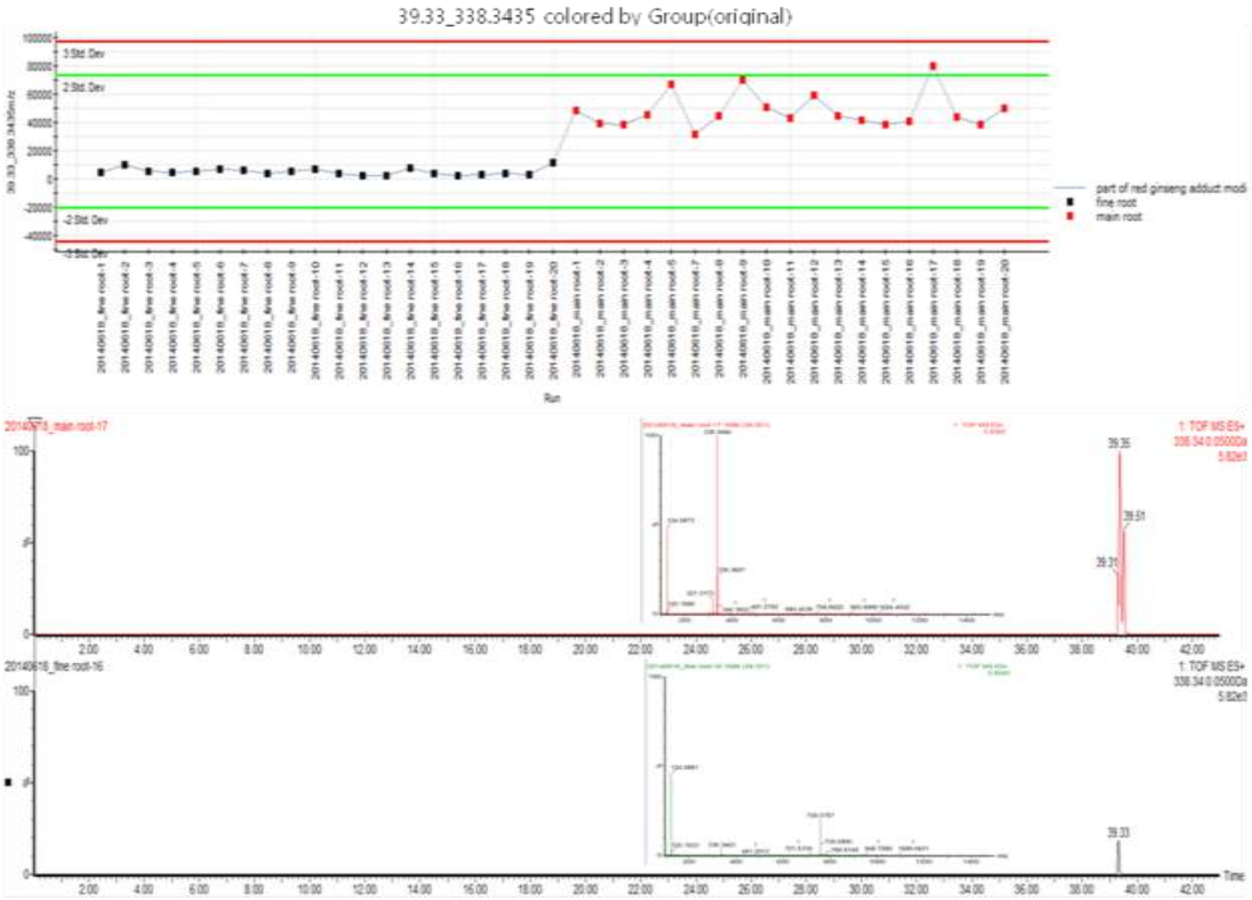

Figure S12. Selected ion intensity trend plots & EIC for ion **1**

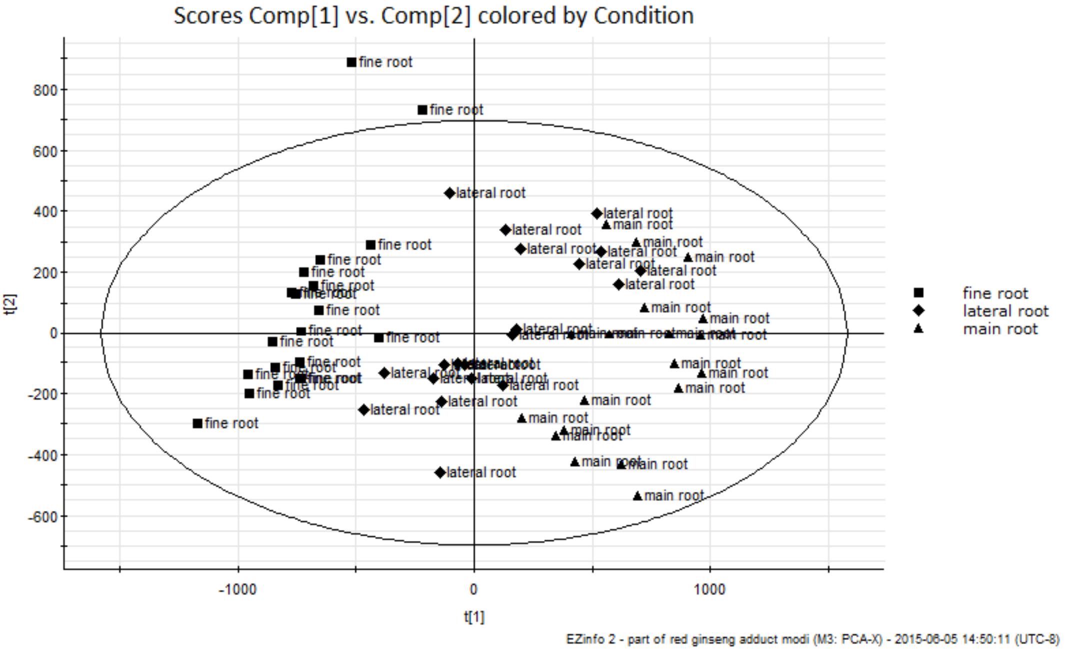

Figure S13. PCA score plot (main root, lateral root, and fine root)

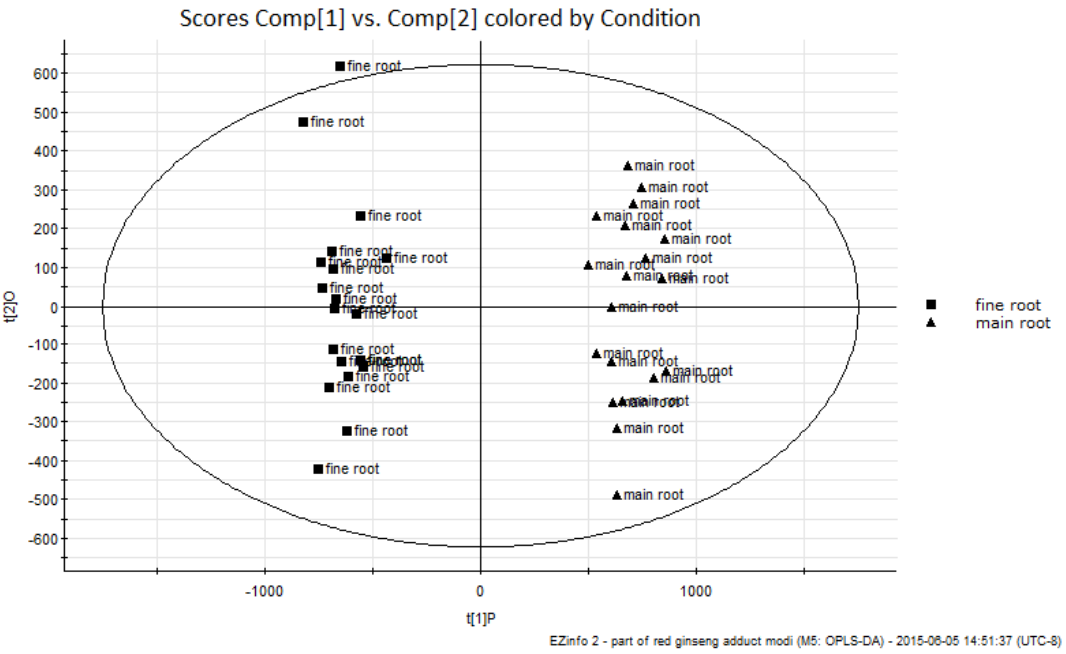

Figure S14. OPLS-DA score plot (main root and fine root)

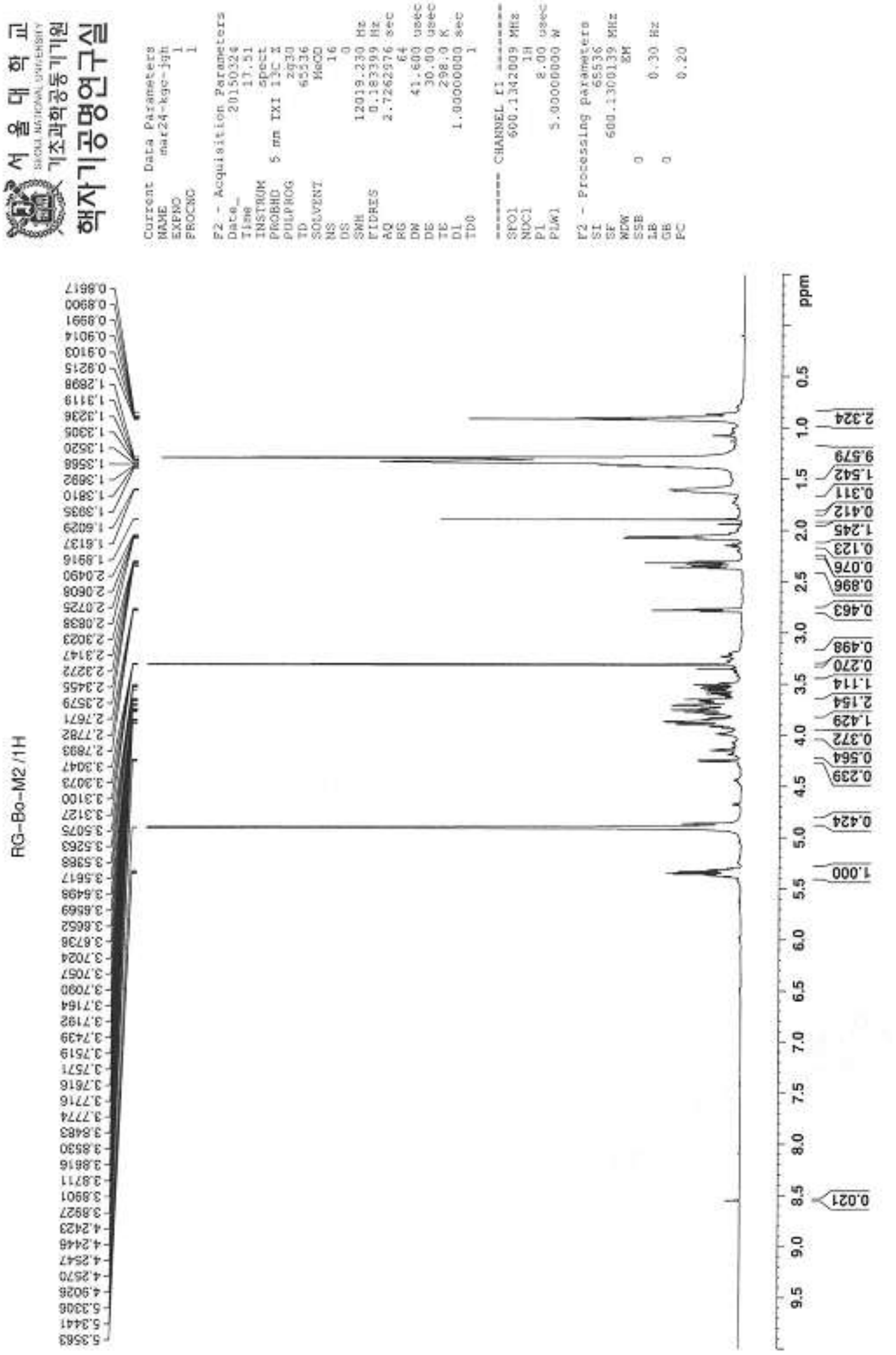

Figure S15. The ^{1}H NMR spectrum of compound **1** (ion **g**)

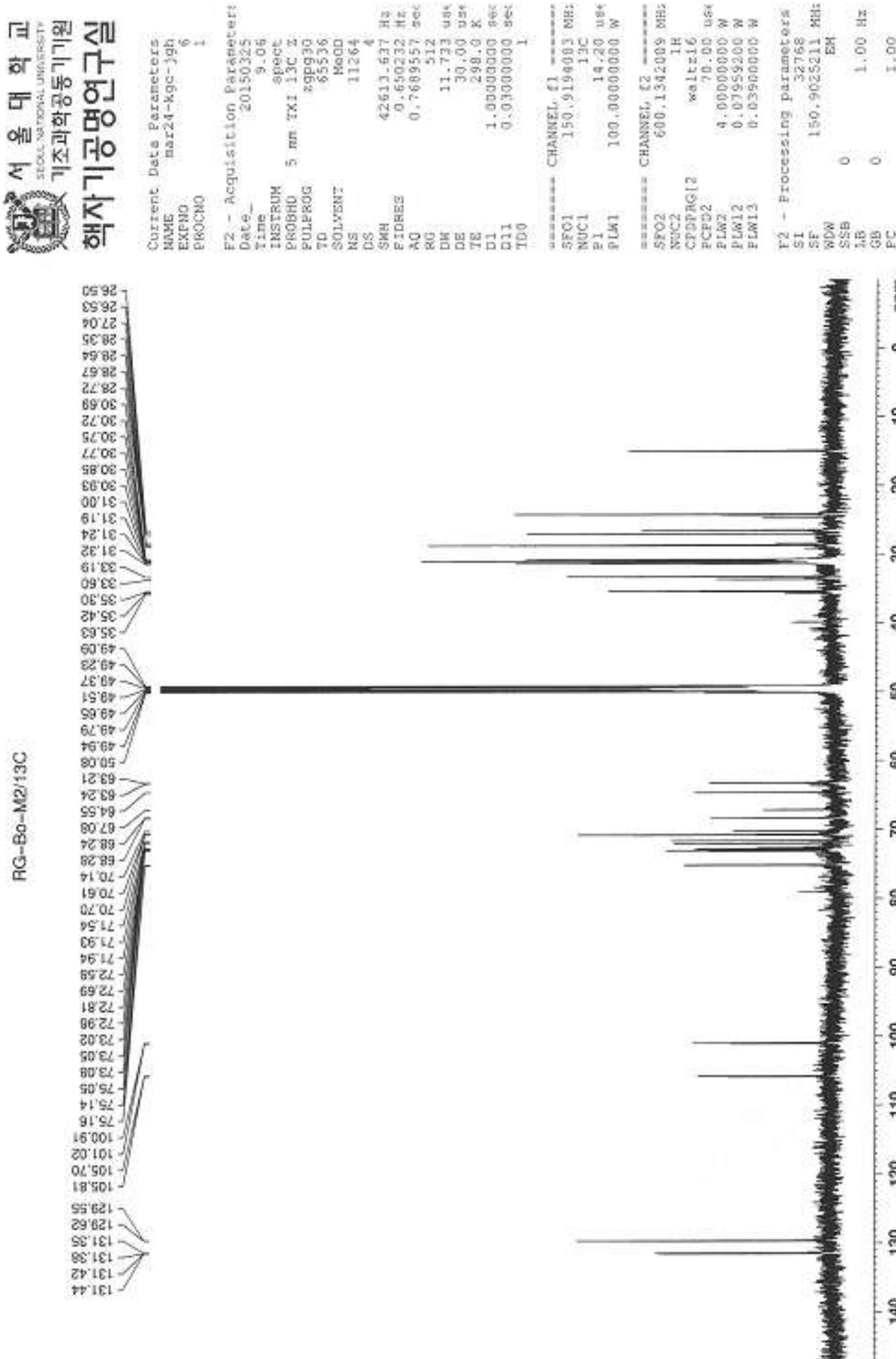

Figure S16. The ^{13}C NMR spectrum of compound **1** (ion **g**)
